# Fetal-Neonatal Ovarian Cysts-Their Monitoring and Management: Retrospective Evaluation of 20 Cases and Review of the Literature

**DOI:** 10.4274/jcrpe.v2i1.28

**Published:** 2010-12-08

**Authors:** Mustafa Ali Akın, Leyla Akın, Sibel Özbek, Gülay Tireli, Sultan Kavuncuoğlu, Serdar Sander, Mustafa Akçakuş, Tamer Güneş, M. Adnan Öztürk, Selim Kurtoğlu

**Affiliations:** 1 Erciyes University Faculty of Medicine, Department of Pediatrics, Division of Neonatology, Kayseri, Turkey; 2 Erciyes University Faculty of Medicine, Department of Pediatrics, Division of Pediatric Endocrinology, Kayseri, Turkey; 3 Bakırköy Maternity and Children Hospital, Department of Pediatrics, Division of Neonatology, İstanbul, Turkey; 4 Bakırköy Maternity and Children Hospital, Department of Pediatric Surgery, İstanbul, Turkey; +90 532 561 79 45mustafaaliakin@hotmail.comErciyes University Faculty of Medicine Department of Pediatrics Division of Neonatology, Kayseri, Turkey

**Keywords:** newborn, Ovarian cyst, management modality

## Abstract

**Objective**: Neonatal ovarian cysts (NOC) are usually self-limiting structures. However, large or complex cysts may lead to severe complications. A standard guide to management, treatment and follow-up of NOC is not yet available. The aim of this study was to evaluate retrospectively the records of NOC patients from two medical centers.

**Methods**: A total of 20 newborns with NOC were included in the study. The size and localization of the cyst, the age, the signs and symptoms at presentation, and the possible maternal and fetal-neonatal etiologic factors were recorded. Follow-up procedures and treatment modalities were evaluated.

**Results**: The mean age at diagnosis was 34 gestational weeks. The cysts (mean size 53±15 mm) were predominantly in the right ovary (75%) and were evaluated as large cysts in 16 (80%) of the patients. In 5 of the patients with large cysts and in 1 of the 4 patients with small cysts, the cysts were evaluated as complex cysts. Torsion of the ovary was detected in five (25%) cases and these cases were treated surgically. Patients with simple cysts were closely followed by ultrasonography until the cysts disappeared.

**Conclusion**: To date, there is no precise guide for the monitoring and treatment of NOCs. Surgical treatment should always be performed in a way to protect the ovaries and to ensure future fertility. In our NOC series, it has been possible to apply a non-invasive follow-up program and minimally invasive surgical procedures.

**Conflict of interest:**None declared.

## INTRODUCTION

Ovarian cysts are the most frequently encountered abdominal tumors in female fetuses and newborns (1). The first neonatal ovarian cyst (NOC) was reported in 1889 as an autopsy finding in a stillborn preterm infant (2). Nowadays, with the advances in radiographic techniques and especially following the extensive use of ultrasonography, NOCs are easily detected in the prenatal period, towards the end of second trimester of gestation (1, 3, 4, 5, 6, 7). 

It is generally accepted that in the newborn, the term ”pathological cyst" refers to one with a diameter over 2 cm (8, 9). The distinction between mature follicle cysts and ovarian cysts is made according to their size only (8, 10). However, in addition to size, the symptoms and the ultrasonographic features of the cysts are needed to determine the treatment modality and prognosis. The presence of ovarian cysts in the fetus and newborn is a sign of an abnormal exacerbation of the physiologic process (8). Small cysts (follicle cysts) occur at a frequency of 90%, while large cysts are reported in 20-34% of newborns who succumb within the first 28 days of life (7, 11). Mature ovarian follicles are seen in 33% to 60% of newborn babies (8). 

Cysts are classified with regard to their ultrasonographic features as “simple” or “complex”, and with regard to their size as “small” or “large” cysts (12, 13). The primary structures of simple cysts are functional and they are also called follicular cysts (5, 11, 14, 15). They are almost always unilocular, and they are seen in microscopic sections as follicle cysts surrounded by granulosa epithelial cells (1, 8). The term “complex cyst” is used for a thick-walled septated cyst which contains blood clot or debris.

Small cysts are usually asymptomatic and showspontaneous regression within a few months after birth (1, 5, 16). Rarely, they are complicated, depending on their size and pedicle length (5, 6, 11, 17). The most frequent and frightening complication of simple cysts is torsion of the ovary. Other complications are intracystic hemorrhage, rupture, dystocia during birth, pressure on nearby structures such as blood vessels, uterus, intestines and urinary system (3, 4, 5, 6, 14, 15, 16, 18). If a cyst is complex from the beginning, there is a probability of ovarian-vascular dysgenesis or of a neoplasm (5, 16, 17). Complex cysts generally occur as complications of large cysts. The treatment modality of NOC, especially of large simple cysts is controversial, while the treatment of torsioned or complicated cysts is unequivocally surgical and causes the loss of the affected ovary. 

The aim of the present study was to evaluate retrospectively the management and follow-up data of 20 cases with NOC from two different medical centers.

## METHODS

A total of 20 patients diagnosed as NOC in the Neonatology Units of two hospitals (Bakırköy Maternity Hospital in Istanbul and Erciyes University Gevher Nesibe Hospital in Kayseri) between the years of 1987 and 2009 were evaluated. Data on 17 patients were obtained from patient records and on 3 patients prospectively. 

Patients who were antenatally diagnosed were followed by ultrasonography twice a month until birth. Ultrasonographic evaluation was repeated within 48 hours of birth. Patients who were postnatally diagnosed were followed twice a month during the first three months, then once a month until the cysts shrank and disappeared.

The cysts were classified according to their size as "small" for cysts smaller than 40 mm in diameter and "large" for those larger than 40 mm. Besides, according to their ultrasonographic features they were classified as "simple" and "complex". The Nussbaum criteria were used for discrimination between complex and simple cysts. According to these criteria, “simple cysts” are completely anechoic, homogeneous, thin-walled, and are frequently unilocular and located unilaterally. Thick-walled cysts having a solid structure and septa, and which contain blood clots and debris are “complex cysts” (12, 13). 

The size and localization of the cyst, the age at which they were detected, the signs and symptoms at presentation were recorded. The maternal and fetal-neonatal etiological factors were also recorded. The birth modality was determined as per obstetric indication. 

The treatment modalities and follow-up procedures were evaluated. All cysts which were evaluated as”complex”, as well as those which were torsioned and symptomatic, were surgically treated, regardless of their size. Histopathological diagnoses of surgically removed materials were compared with ultrasonographic findings.

## RESULTS

Eighteen (90%) of 20 patients were diagnosed antenatally. The age of antenatal diagnosis ranged between 34 and 38 gestational weeks. The mothers of 2 postnatally diagnosed cases had not undergone antenatal ultrasonographic examination during the last five months of their pregnancy. These two patients were referred to our clinics for abdominal mass detected at routine check-ups during the first postnatal week. 

Gestational diabetes was detected in 5 mothers and pre-eclampsia in 2. Polyhydramniosis was present in all cases. Rh incompatibility without hydrops was noted in one patient. Thyroid function tests were within normal ranges in all patients. No patient had any congenital malformations. 

The cysts were unilateral and detected in the right ovary in 15 (75%) of patients. There were no cases with bilateral cysts. The mean size of the cysts was 53.0±15.0 mm (range 25mm and 80mm). Sixteen cases (80%) had large cysts and four cases (20%) small cysts. Simple cysts were detected in 14 (70%) cases, and complex cysts in 6 cases (30%). Five of the 16 cases with large cysts were complex due to ovarian torsion. All of the torsions were detected in the antenatal period. Ovarian torsion was detected in two (40%) of five cases with no clinical symptoms. Two of the patients without ovarian torsion presented with a sign other than abdominal mass; one with hydronephrosis, and the other one with intestinal obstruction. One of the 4 cases with small cysts was also complex due to intracystic hemorrhage. Demographic, clinical, ultrasonographic and other features of the patients are given in Table 1. 

As shown in Table 2, the cases with simple cysts were treated surgically. Two of these patients underwent cystectomy, six-total oophorectomy, and three-salpingo-oophorectomy. Cyst aspiration was not performed in any of the fetuses. The remaining 3 of the 14 cases with simple cysts were closely followed by ultrasonography until the cysts shrank and disappeared. All of these cases spontaneously resolved within three to six months. The six cases with cysts with torsion (complicated) were treated with salpingo-oophorectomy except for one, who underwent oophorectomy (Table 1). 

Histopathological diagnostic studies of the surgically removed cysts revealed that simple cysts were follicular or techa-lutein cysts and torsion-complex cysts were hemorrhagic. No cysts were noted to have malignant features.

**Table 1:**
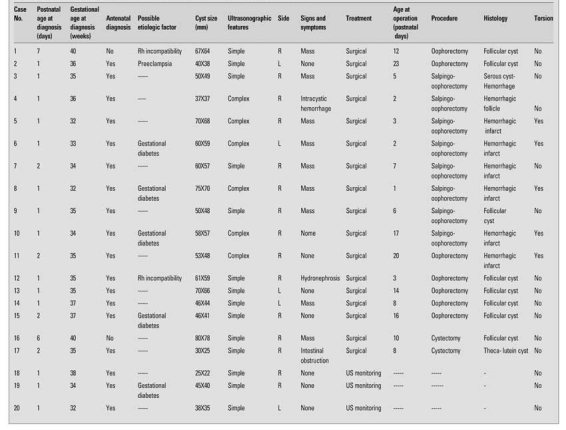
Demographic, clinical, ultrasonographic and other features of our patients with ovarian cysts

**Table 2:**
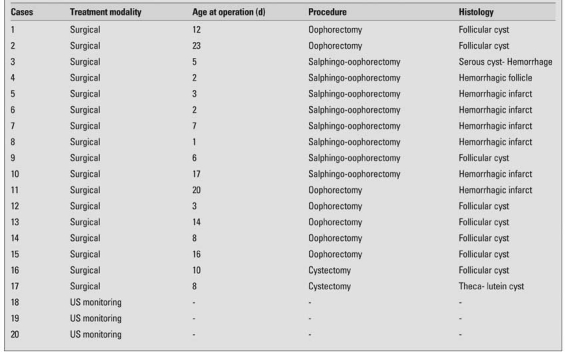
Management and histopathological features of the cysts in our cases

## DISCUSSION

Neonatal ovarian cysts are nearly always benign and self-limiting, and many of them go unreported (8, 11, 16). In girls, the frequency of such cysts was reported as approximately 5% of all abdominal masses in the first month of life (17).

The first prenatal detection of a fetal ovarian cyst was in 1975 by Valenti (12, 18). Nowadays, NOCs may be detected by antenatal ultrasonography towards the end of the second trimester of gestation. The 19^th^ week of gestation is the earliest age at which NOC was detected, but most cases are detected after 28 gestational weeks (1, 11, 14, 15). Most of our cases (90%) were detected during the antenatal check-ups between 32-36 gestational weeks. The mean size of the antenatally diagnosed cysts in our series was 50.7±13.9 mm and minimum the size was 25 mm. The size of all cysts remained unchanged until birth, unless they torsioned and became hemorrhagic. When cysts are once detected, close ultrasonographic follow-up is necessary in order to detect possible complications and to make a differential diagnosis (5, 11). Mesenteric, omental and urachal cysts, duplication anomalies, structures or anomalies leading to intestinal or urinary obstruction such as renal cysts, cystic meconium peritonitis, hydrometrocolpos, duodenal atresia, as well as anterior meningocele should be considered in the differential diagnosis of a cystic abdominal mass in a female fetus. Malignant tumors are rare in the neonatal period, but benign cystic teratomas are the most common ovarian tumors (11, 14, 15, 16, 19). Lymphangiomas also are counted among the hamartomatous lesions of the fetal-neonatal ovary (16, 19). 

In NOC cases, chromosomal and congenital malformations are absent (8, 11, 15). The incidence of NOC rises with increasing placental chorionic gonadotropin levels in complicated pregnancies with large placenta such as in diabetes, pre-eclampsia and Rh incompatibility (1, 5, 6, 7, 8, 13, 14, 15, 16). Another endocrinologic cause of NOC in preterm babies is the immaturity of the gonadostat mechanism, which causes ovarian hyperstimulation (5, 16). Additionally, fetal hypothyroidism and congenital adrenal hyperplasia due to 21-hydroxylase deficiency or 11 beta-hydroxylase deficiency have also been reported to cause NOC (1, 6, 8, 16, 20). The decrease in maternal-placental estrogens and β-hCG after birth and the baby’s neurologic maturation lead to spontaneous regression in the cysts. However, since FSH-LH levels of infants continue to increase until the maturation of the gonadostat mechanism, cysts may continue to enlarge for about three months after birth (5, 6, 15, 16). Despite all this knowledge, the definite causes of NOC remain unclear. The possible etiologic factors in our cases were as follows: five cases (25%) were infants of diabetic mothers, two cases (10%) were the offspring of mothers with pre-eclampsia, and one case (5%) had Rh incompatibility. Thyroid hormone levels were normal in all cases. No congenital anomalies were detected. 

Spontaneous regression occurs in more than half of NOC cases in the prenatal or postnatal period. The majority of these cases are small and simple cysts (1). In our cases, of the 14 patients with simple cysts who were followed with serial ultrasonography, three (75%) showed spontaneous resolution within six months at the latest check-up. In one patient, which initially had a small sized cyst, the cyst rapidly enlarged and acquired complex features with hemorrhage in postnatal life. Of the 14 cases with simple cysts, 5 (35%) changed to complex cysts owing to ovarian torsion. However, hemorrhage developed in one (7%) simple-small cyst without ovarian torsion. 

Neonatal ovarian cysts may cause pain, irritability, vomiting, fever and abdominal distension. Peritonitis, anemia due to intracystic hemorrhage, fetal tachycardia due to peritoneal irritation or anemia, and sudden infant death syndrome may also occur (5, 7, 13, 14, 15, 16). The large cysts may cause intestinal and urinary obstruction due to their size, dystocia, and therefore abdominal and thoracic mass effect gives rise to pulmonary hypoplasia and polyhyramniosis (5, 15, 16). When a cyst is torsioned, its size rapidly increases and its ultrasonographic features change to complex. The ovarian torsion frequently leads to further complications such as rupture resulting in hemoperitoneum, ascites stemming from transudation, adhesion with adjacent organs resulting in urinary and intestinal obstruction, calcification of cyst walls, and auto-amputation of the ovary. On abdominal examination, a mobile mass can be palpable if auto-amputation of a torsioned ovary occurs (1, 3, 4, 5, 6, 7, 8, 14, 15, 16). 

When the small cysts start to grow rapidly and their ultrasonographic features change, ovarian torsion should be considered (1, 8, 17). Ovarian torsion is the most frequent complication (%25-75) of NOC, and mostly occurs in large cysts (8, 16). The risk of ovarian torsion is related more to the length of the pedicle than to the size of the cyst (1, 8, 15, 16, 17, 18). Ovarian torsion, rare in the postnatal period, is relatively more frequent in the intrauterine period and during birth (6, 8, 16). Ovarian torsion and necrosis are followed by intracystic hemorrhage or vice versa (4, 5, 6, 16). Ovarian cysts which are complex all along may result in a disrupted vascularization of the primitive gonad and the ipsilateral fallopian tube is often atretic (17). Ultrasonography is usually sufficient to determine ovarian torsion, but sometimes MRI is also necessary to determine the age of the hemorrhage (1, 5, 13). Ovarian torsions were encountered in five of our cases, all during the intrauterine period. Two of the patients with ovarian torsion had no symptoms. 

 No change in the method of delivery is necessary after prenatal identification of a cyst (1, 6, 8, 11). Today, regardless of the size of cysts and their ultrasonographic features, unless an obstetric indication is present, vaginal delivery is recommended (6, 13, 16). In our series, in the 18 patients identified during the antenatal period, the average size of the cysts was 50.7±13.9 mm. Seven of these babies (40%) were born by cesarian section (C/S) and the remaining by vaginal delivery. Pre-eclampsia and previous C/S deliveries were stated as the indications for C/S. 

**Figure 1:**
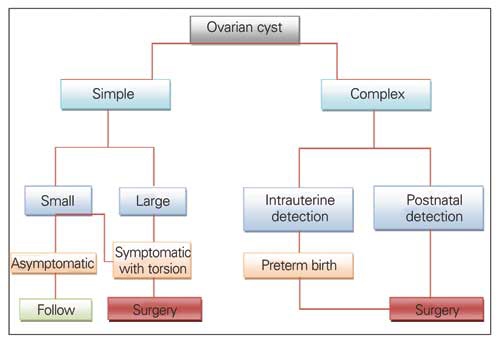
Management approach for fetal-neonatal ovarian cysts

There is no concensus in the modality and timing of the treatment or monitoring of NOC. Current information regarding the treatment and follow-up of NOC is based on personal experiences and some small case series. However, it is certain that surgical treatment is essential in symptomatic, complicated and torsioned cysts (6, 8, 16, 18). The major goal of both surgical treatment and non-invasive monitoring by US is optimal ovarian preservation. However, long-term outcome and risk to future fertility is unknown (3, 15). As long as simple cysts are small, do not show a trend for rapid growth and remain asymptomatic, they should be monitored by serial ultrasonography during the prenatal period and also postnatally. In the postnatal period, if a cyst is large, does not regress or increases in size, surgical intervention is recommended to prevent complications such as ovarian torsion and bleeding (3, 6, 8, 18). Ultrasound-guided cyst aspiration is recommended for simple cysts which are larger than 40 mm, since they may cause pressure on the neighboring fetal organs. However, the risk of recurrence is high. Moreover, this intervention carries potential risks such as cyst rupture, peritonitis, preterm labor, chorio-amnionitis, and fetal injury (1, 3, 6, 11, 15). Aspiration of an ovarian cyst during fetal life is recommended only if the cyst is large enough to impair spontaneous delivery or cause distension of the fetal abdomen (1, 5, 15). Postnatal percutaneous aspiration of NOC has also been proposed for large cysts to reduce the risk of ovarian torsion and other complications (15, 21). In the light of this growing knowledge we propose the use of an algorithm, such as that presented in Figure 1 for the management of fetal-neonatal ovarian cysts. In our patients who had simple cysts initially, three underwent salphingo-oophorectomy, two - cystectomy, and six-total oophorectomy. No fetus underwent intrauterine cystaspiration. 

Complex cysts (mostly torsioned simple cysts) cannot be distinguished from other intra-abdominal pathology and require surgical exploration. In fetal life, when intracystic hemorrhage and ovarian torsion are detected, preterm birth is necessary after lung maturity has been attained (4). Surgical intervention should be minimally invasive and should aim to save the ovary. Minimally invasive approaches such as laparoscopic and microendoscopic approaches are recommended (1, 3, 4, 5, 6, 7, 8, 10, 11, 12, 13). The laparoscopic approach is to be preferred since it affords a diagnostic opportunity by showing both ovaries. Additionally, it allows aspiration of the cyst, cystectomy, decapsulation of the ovary, stripping of cysts and, if necessary, oophorectomy (7, 8, 9, 10, 16, 19). All of the five patients with ovarian torsion in our series underwent salpingo-oophorectomy, and one patient with a complex cyst without ovarian torsion underwent oophorectomy. The histopathological study of all torsioned cysts revealed hemorrhagic infarcts. The one patient with the complex cyst was histopathologically diagnosed as a hemorrhagic cyst. 

In conclusion, ovarian cysts are seen more frequently than expected in the neonatal period and they can be life-threatening because of their certain complications. To date, there is no precise guide for the monitoring and treatment of NOCs. Surgical treatment should always be performed in a way to protect the ovaries and to ensure future fertility. In our NOC series, it has been possible to apply a non-invasive follow-up program and minimally invasive surgical procedures.
